# Gut Microbiome Distinguishes Patients With Epilepsy From Healthy Individuals

**DOI:** 10.3389/fmicb.2021.696632

**Published:** 2022-01-07

**Authors:** Guangying Cui, Shanshuo Liu, Zhenguo Liu, Yuan Chen, Tianwen Wu, Jiamin Lou, Haiyu Wang, Yawen Zou, Ying Sun, Benchen Rao, Zhigang Ren, Yajun Lian, Yan Jiang

**Affiliations:** ^1^Department of Infectious Diseases, First Affiliated Hospital of Zhengzhou University, Zhengzhou, China; ^2^Gene Hospital of Henan Province, Precision Medicine Center, First Affiliated Hospital of Zhengzhou University, Zhengzhou, China; ^3^Department of Neurology, First Affiliated Hospital of Zhengzhou University, Zhengzhou, China

**Keywords:** epilepsy, gut microecosystem, microbial biomarkers, operational taxonomic unit (OTU), fecal microbiome

## Abstract

**Objective:** The gut microecosystem is the largest microecosystem in the human body and has been proven to be linked to neurological diseases. The main objective of this study was to characterize the fecal microbiome, investigate the differences between epilepsy patients and healthy controls, and evaluate the potential efficacy of the fecal microbiome as a diagnostic tool for epilepsy.

**Design:** We collected 74 fecal samples from epilepsy patients (Eps, *n* = 24) and healthy controls (HCs, *n* = 50) in the First Affiliated Hospital of Zhengzhou University and subjected the samples to 16S rRNA MiSeq sequencing and analysis. We set up a train set and a test set, identified the optimal microbial markers for epilepsy after characterizing the gut microbiome in the former and built a diagnostic model, then validated it in the validation group.

**Results:** There were significant differences in microbial communities between the two groups. The α-diversity of the HCs was higher than that of the epilepsy group, but the Venn diagram showed that there were more unique operational taxonomic unit (OTU) in the epilepsy group. At the phylum level, *Proteobacteria* and *Actinobacteriota* increased significantly in Eps, while the relative abundance of *Bacteroidota* increased in HCs. Compared with HCs, Eps were enriched in 23 genera, including *Faecalibacterium*, *Escherichia-Shigella*, *Subdoligranulum* and *Enterobacteriaceae-unclassified*. In contrast, 59 genera including *Bacteroides*, *Megamonas*, *Prevotella*, *Lachnospiraceae-unclassified* and *Blautia* increased in the HCs. In Spearman correlation analysis, age, WBC, RBC, PLT, ALB, CREA, TBIL, Hb and Urea were positively correlated with most of the different OTUs. Seizure-type, course and frequency are negatively correlated with most of the different OTUs. In addition, twenty-two optimal microbial markers were identified by a fivefold cross-validation of the random forest model. In the established train set and test set, the area under the curve was 0.9771 and 0.993, respectively.

**Conclusion:** Our study was the first to characterize the gut microbiome of Eps and HCs in central China and demonstrate the potential efficacy of microbial markers as a noninvasive biological diagnostic tool for epilepsy.

## Introduction

The fecal microflora, as a major component of the gut microecosystem, plays an important role in human health and diseases and is also a significant regulator of environmental factors affecting host disease risk. The gut microbiome is closely related to and interacts with various organs and systems of the human body, including the brain, lungs, liver, bones, and cardiovascular system (Feng et al., 2018). As sequencing and metabolomics technologies have improved and costs have decreased, gut microbes have received more attention and research. A growing body of clinical and preclinical evidence indicated that gut microbiota regulated the development and homeostasis of the central nervous system through immune, circulatory, endocrine and neural pathways (Tremlett et al., 2017; Lum et al., 2020), which played a key role in neuropsychiatric disorders such as anxiety, depression, autism, Alzheimer’s disease, multiple sclerosis, Parkinson’s disease, and stroke (Benakis et al., 2016; Minter et al., 2016; Sampson et al., 2016; Foster et al., 2017; Fung et al., 2017). In a study of infant neurodevelopment (Carlson et al., 2018), cognitive function at age 2 was significantly associated with the composition of the microbiota 1 year earlier. Experiments in germ-free mice also demonstrated that basic neural processes depend on the composition of the microbiota (Luczynski et al., 2016). Gut microbiome may also play a key role in aging and neurodegeneration by modulating microglial activation (Erny et al., 2015).

Epilepsy is a chronic neurological disease characterized by recurrent seizures. Epidemiological statistics have indicated that more than 50 million people worldwide suffer from this disease (Perucca et al., 2020), which is associated with significant rates of healthcare costs. The prevalence of epilepsy in China has more than doubled in the past 20 years (Song et al., 2017), and it is estimated that more than 2.4 million people suffer from epilepsy annually. Both genetic and environmental factors can influence an individual’s susceptibility to epilepsy, but the exact causes in most people with epilepsy are unknown. In addition, approximately 30% of Eps are resistant to traditional antiepileptic drugs, indicating that the need for new treatments and biomarkers for epilepsy research remains unmet (Chen et al., 2018). According to the Chinese Guidelines for the Clinical Diagnosis and Treatment of Epilepsy, the novel definition of epilepsy has three elements: at least one seizure; a tendency and susceptibility to recurrent seizures; and the corresponding neurobiological, cognitive, psychological and social effects and obstacles. A detailed clinical history and a reliable eyewitness account of seizures are the basis for diagnosis. This diagnosis is determined by a series of clinical characteristics, including age of onset, seizure types, comorbidity, EEG, and imaging features (Thijs et al., 2019). Among them, EEG is a very key diagnostic tool, seizures are often accompanied by abnormal EEG performance, and when it can’t capture habitual seizures, interictal epileptiform discharges, such as spikes and sharp waves, are often used to support the diagnosis of epilepsy. However, the absence of interictal epileptiform discharges in conventional EEG does not exclude the possibility of epilepsy, and only about 50% of patients with a history of epilepsy had epileptiform discharges in conventional tests (Chen and Koubeissi, 2019). This method, which relies on subjective description and does not provide adequate warning, has a very limited effect on the onset and prevention of epilepsy. The complex etiology of epilepsy and the lack of reliable human biomarkers have forced us to urgently seek new strategies.

In recent years, with the development of sequencing technology, the relationships between the composition, function and metabolic potential of the microbiome and diseases have been extensively studied. The diagnostic potential of the gut microbiome for a variety of neurodevelopmental, neuropsychiatric, and neurodegenerative diseases has been confirmed by compelling studies, but only a few studies have highlighted the distinctions between the fecal microbiota in Eps and HCs, finding that the intestinal microbiota may play an important role in epileptic seizures and thus may represent a new target for drug therapy or a biomarker (Xie et al., 2017; Peng et al., 2018; Lindefeldt et al., 2019), the diagnostic potential of the gut microbiome for epilepsy needs to be further evaluated. The objectives of this study were to investigate the differences in fecal microbial composition between Eps and HCs and to verify the efficacy of fecal microorganisms as a diagnostic tool for epilepsy using several detected biomarkers.

## Materials and Methods

### Participant Inclusion and Exclusion Criteria

The study was designed and performed in accordance with the PRoBE (prospective specimen collection and retrospective blinded evaluation) design, the Helsinki Declaration, and the Rules of Good Clinical Practice (Ren et al., 2019). Before commencing the experiments, ethical clearance was sought from the First Affiliated Hospital of Zhengzhou University, and the study was approved (No. 2021-KY-0574-002). Written informed consent was obtained from each enrolled participant.

A total of 24 patients with epilepsy from the outpatient department of the First Affiliated Hospital of Zhengzhou University were enrolled from June 2019 to October 2019, and fecal samples were collected prospectively. Patients were included if they met the following criteria: (1) met the 2005 ILAE criteria for epilepsy; (2) had at least one epileptic seizure without a fixed cause; (3) had a tendency toward recurrent attacks; and (4) were first diagnosed as having epilepsy. Participants with the following conditions were excluded: (1) other neurological conditions similar to epilepsy; (2) use of antibiotics or probiotics in the past 3 months; and (3) a known history of chronic illness; (4) pregnant and lactating women.

Fifty HCs from the physical examination department of the First Affiliated Hospital of Zhengzhou University were also enrolled in this study. Inclusion criteria: (1) good physical condition, no special discomfort symptoms compared with usual; (2) mentally normal, with normal expression ability; (3) volunteer to participate in this study; Exclusion criteria: (1) complicated with malignant tumors, serious cardiovascular and cerebrovascular diseases, infectious diseases and other mental and nervous system diseases; (2) younger than 18 years of age or older than 80 years of age; (3) pregnant and lactating women; (4) lack of relevant clinical information; (5) use of antibiotics or probiotics in the past 3 months. Clinical information including gender, age, seizure frequency, seizure types and clinical features was registered prospectively. Fecal samples were prospectively collected from the enrolled participants and subjected to 16S rRNA MiSeq sequencing.

### Fecal Sample Collection and DNA Extraction

Fresh stool samples were collected from all participants. Each stool sample was subjected to routine testing to assess stool consistency (Vandeputte et al., 2017). The sample was divided into five aliquots of 200 mg, placed in a DNA preservation tube following the protocol recommended by the manufacturer, and immediately stored in a −80° freezer within 2 h. A QIAamp Fast DNA Stool Mini Kit (Qiagen, Hilden, Germany) was used to perform the DNA extraction (Tang et al., 2018).

### Polymerase Chain Reaction Amplification and MiSeq Sequencing

Extracted DNA was amplified with PCR in a 20-μL reaction system. The V3-V4 hypervariable region of 16S rRNA was targeted by the forward primer 5′-CCTACGGGNGGCWGCAG-3′ and reverse primer 5′-GACTACHVGGGTATCTAATCC-3′. PCR was conducted in an ABI Gene AmpR 9700 system (Thermo Fisher Scientific, Waltham, MA, United States). Specifically, PCR included 2 min at 95°C followed by 30 cycles of denaturation (95°C for 30 s), annealing (55°C for 30 s), and extension (72°C for 30 s) and a final extension at 72°C for 5 min (Ren et al., 2019). Samples were collected and subjected to quality control by electrophoresis on a 2% (w/v) agarose gel, and we used AxyPrep™ DNA gel (Axygen Scientific, Waltham, MA, United States) and a PCR Cleanup System (Promega, Madison, WI, United States) to extract and purify the bands. The amplicons were then sequenced on the Illumina HiSeq PE250 platform. The purified PCR products were mixed, and a DNA library was constructed according to the manufacturer’s instructions. Sequences were merged with the Illumina MiSeq platform by Shanghai Mobio Biomedical Technology Co. Ltd., Shanghai, China (Ren et al., 2013), and raw Illumina read data were deposited in the European Nucleotide Archive Database of the European Bioinformatics Institute under accession number PRJNA 701117.

### Sequence Data Processing

The filtered reads were distributed to different samples based on specific barcodes, and then the barcodes and primers were removed. The paired-end sequenced reads of each library were overlapped with the default parameters using FLASH v. 1.2.10 (Magoč and Salzberg, 2011). The combined sequence was obtained, and the original data were quantitatively analyzed for quality control. The overlapping reads generated by FLASH were subjected to quality control to filter out mismatches in the barcode/primer region, ambiguous bases, and reads with >5 in the overlap region. Briefly, the sequences with an average quality score less than 20, more than one ambiguous base, and a length less than 220 bp or more than 500 bp were filtered out. The read data were demultiplexed and assigned to different samples according to the barcodes. Chimeric sequences were also detected and removed using UCHIME v. 4.2.40 (Edgar et al., 2011). All remaining sequences were then clustered into operational taxonomic units (OTUs) according to sequence similarity after singleton removal. The Broad Institute 16S “gold standard” database was used as a reference (Microbiome, Util - r20110519 version^1^) to match the operational classification units (OTUs).

### Operational Taxonomic Unit Clustering and Taxonomic Annotation

Equal numbers of reads were randomly selected from all the samples; among them, a large number of sequences and singletons were sifted out. The unique sequences were clustered into OTUs by using the “usearch-CLUster-otus” command, the selected sequence was compared with the OTU sequences by using the “usearch-usearch-global-ID 0.97” command, and the identity threshold was set to 0.97. Then, the OTU composition table was created (Lu et al., 2019). After the OTUs were binned with the UPARSE pipeline (Edgar, 2013), we counted the gross OTUs at each taxonomic level (phylum, class, order, family, and genus). The results are presented in a statistical table listing the OTU sequence numbers of each sample.

### Bacterial Diversity and Taxonomic Analysis

A rarefaction curve was plotted to compare microbial community richness among the samples and validate the sequencing data. Similarly, a Shannon–Wiener curve and rank-abundance curve were used to verify the data quality of the samples. The Venn program was used to identify the overlap and uniqueness of OTUs in the two groups. The within-sample diversity (i.e., α-diversity) was calculated by the Chao1 index, ACE index, Shannon index and Simpson index using the R program package ‘vegan.’ Distance matrices (i.e., β-diversity) were obtained by both the weighted and unweighted unifrac distance metrics and visualized by PCoA. PCoA, which was conducted in R^2^, is functionally similar to NMDS analysis. Unifrac uses phylogenetic information to assess community differences between samples. Weighted unifrac takes the abundance of sequences into account, whereas unweighted unifrac does not. The Wilcoxon rank-sum test R function was used for statistical comparisons of the two groups. Dominant species heat map was plotted with Heat map Builder. Microbial community bar plots were generated by species composition analysis. We compared the sequences with MUSCLE and used Fast Tree MP to generate an unrooted phylogenetic tree with a generalized time-reversible (gtr) model. The phylogenetic tree was rerooted by using a custom Perl script furnished by Microbes Online (reroot.pl)^3^.

Bacterial classification, analysis and comparison were conducted between the EP group and the HC group at the phylum, class, order, family and genus levels. The bacterial compositions in individual samples and the two groups were both plotted. The Wilcoxon rank-sum test was also used to compare the epilepsy group and the HC group. LDA was conducted using the LEfSe method to characterize the fecal microbiomes. The LEfSe method combined both statistical significance (Kruskal test and Wilcoxon test) and LDA to measure the magnitude of differentiation between groups. The threshold LDA score for discriminative features was 3.0 [(log10) = 3]. The LEfSe method and LDA were used to characterize the fecal microbiota, and LDA was used to screen key microbial markers.

### Gene Function Prediction

Phylogenetic Investigation of Communities by Reconstruction of Unobserved States (PICRUSt) was used to predict the metabolic functions of the bacterial flora and 16S rRNA gene sequences in the KEGG, COG, and Rfam databases. Data were then entered into the HUMAnN to find significant differences in KEGG ortholog (KO) abundances. This approach predicts the metabolic functions of bacteria and ancient bacteria by comparing the 16S rRNA gene sequencing data against a reference database of microbial genomes with known metabolic functions.

The annotation information corresponding to each functional spectrum database per sample and the abundance matrix for the predicted functional groups may be obtained from the prediction results of PICRUSt. Relative differences in 16S rRNA gene copy number among species were considered during the prediction process. The original species abundance data were corrected to enhance prediction accuracy and reliability (Wang et al., 2018). The biological metabolic pathway analysis database (KEGG PATHWAY Database) is the core of the KEGG database, in which metabolic pathway categories include Metabolism, Genetic Information Processing, Environmental Information Processing, Cellular Processes, Organismal Systems, and Human Diseases.

### Operational Taxonomic Unit Biomarker Identification and Probability of Disease Determination

A random forest model was used to select significantly different OTUs in each sample group. The generalization error was estimated by a fivefold cross-validation. An OTU frequency profile was generated by mapping reads from the Eps and HC groups onto these represented sequences (Fouhy et al., 2019). A cross-validation error curve was plotted after a fivefold cross-validation. The cutoff point was that with the lowest cross-validation error. The sum of the minimum error and the SD at the corresponding point was defined as the cutoff value. All sets of OTU markers with errors below the cutoff value (≤30) are listed. The optimal set with the fewest OTUs, which revealed the differences between the two groups with the highest accuracy, was identified.

Subsequent analyses, such as ROC analysis, were then performed. Statistical significance was determined with a Wilcoxon rank-sum test (*P* < 0.05) (Deschasaux et al., 2018). The POD index was defined as the ratio of the number of randomly generated decision trees predicting samples as “Epilepsy” to that of HCs. The ROC curve was plotted to evaluate the diagnostic efficacies of the selected biomarkers, and the AUC was also calculated using pROC (R 3.8.1) (Tilg et al., 2018).

### Statistical Analysis

SPSS v. 20.0 (IBM Corp., Armonk, NY, United States) was used to process the data. The statistical significance of the differences between groups was calculated. Wilcoxon rank-sum tests were conducted to compare the continuous variables between groups. Fisher’s exact test was used to compare categorical variables between groups. Spearman’s rank test was used for the correlation analysis.

## Results

### Characteristics of the Participants

**FIGURE 1:**
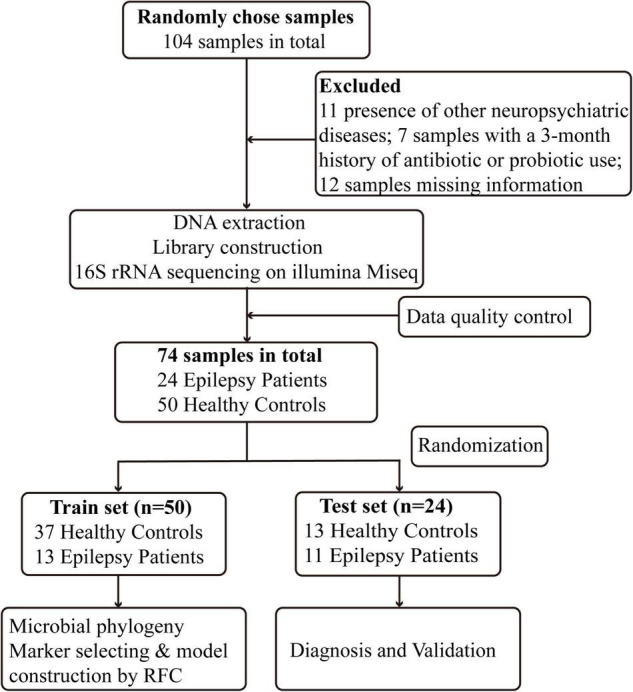
Study design and flow diagram. A total of 104 fecal samples from Central China were collected. After a strict diagnosis and exclusion process, 24 patients with epilepsy and 50 healthy controls were included and randomly divided into the train set (*n* = 50) and test set (*n* = 24). In the train set, we characterized gut microbiome among 13 epilepsy patients and 37 healthy controls and identified microbial markers and constructed epilepsy classifier by random forest model. In test set, 13 healthy controls and 11 epilepsy patients were used to validate diagnosis efficacy of epilepsy classifier. RFC, random forest classifier.

After rigorous screening and exclusion, a total of 74 participants were enrolled, including 24 Eps and 50 HCs. The process and flow chart are shown in Figure 1. The Eps were mainly men, and their seizure types mainly included the following: (1) simple partial seizures (conscious patients, with seizures mainly manifested as paroxysmal paresthesia or local muscle twitches); (2) complex partial seizure (patients exhibited fuzzy consciousness and performed some activities without purpose); and (3) overall stiffness and clonus (the clinical symptoms of the patients were loss of consciousness, limb tics and foaming at the mouth). Moreover, we divided the participants into four grades according to the frequency of seizures within 3 months: grade 1 (0–4 times), grade 2 (5–12 times), grade 3 (13–52 times), and Grade 4 (>52 times). Fecal samples were collected prospectively. The baseline clinical characteristics of the two groups are shown in Table 1.

**TABLE 1:** Characteristics of the participants in this study.

Characteristics	Epilepsy (*n* = 24)	Healthy controls (*n* = 50)	*P*-Value
**Age, mean (±SD)**	30.21 (15.53)	30.1 (5.72)	0.358
**Gender**			
Female, *n* (%)	7 (29%)	15 (30%)	0.941
Male, *n* (%)	17 (71%)	35 (70%)	
**Seizure type (*n*, %)**			
Generalized	17 (71%)	–	–
Simple partial seizure	3 (12.5%)	–	–
Complex partial seizure	4 (16.7%)	–	–
**Frequency (seizures per 3 months) (*n*, %)**			
0–4	16 (66.7%)	–	–
5–12	0 (0%)	–	–
13–52	2 (8.3%)	–	–
>52	6 (25%)	–	–
**Clinical index, mean (±SD)**			
WBC	6.09 (1.86)	5.52 (1.08)	0.959
RBC	4.41 (0.44)	4.90 (0.49)	<0.0001
Hb	135.29 (15.45)	146.55 (16.10)	0.009
PLT	214.36 (42.49)	239.1 (30.75)	0.009
ALB	45.47 (5.67)	47.49 (3.95)	0.001
UREA	4.53 (1.62)	4.85 (1.30)	0.137
CREA	63.26 (14.45)	75.11 (12.93)	0.001
UA	269.74 (96.34)	306.42 (83.07)	0.074
TBIL	8 (4.40)	9.39 (5.33)	0.299

*SD, standard deviation; WBC, white blood cells; RBC, red blood cells; Hb, hemoglobin; PLT, platelets; ALB, albumin; UREA, urea; CERA, creatinine; UA, uric acid; TBIL, total bilirubin.*

### Data Quality and Intestinal Microbial Diversity in Patients With Epilepsy

**FIGURE 2:**
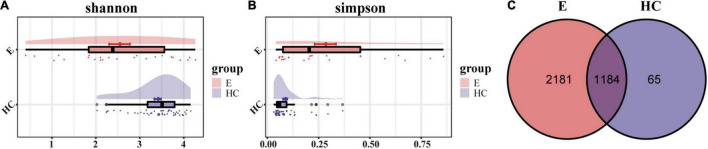
α-Diversity in the fecal microbiota of Eps (*n* = 24) and HCs (*n* = 50). **(A)** The Shannon index was higher in HCs, indicating that the α-diversity of HCs was higher. **(B)** The higher the Simpson index is, the lower the microbial diversity. Therefore, the Eps had a lower diversity than the HCs. **(C)** The Venn diagram displaying overlap between the two groups showed that 1184 of the 3430 OTUs were shared between the Eps and HCs (blue). A total of 2181 of the 3430 OTUs were unique to Eps. Eps, patients with epilepsy; HCs, healthy controls; OTUs, operational taxonomic units.

The rank-abundance curve and Shannon–Wiener curve showed species diversity in epilepsy group and HC group. The curve was smooth and the species uniformity was good. However, the rarefaction curve and the species accumulation (Specaccum) curve showed that the sample size of the epilepsy group was small, and it was better to increase the sample size for more complete studies (Supplementary Figure 1). We measured α-diversity in two groups through five indicators, which mainly reflected the number, abundance and evenness of species in the samples (Supplementary Data 1). According to Shannon and Simpson index, gut microbial diversity in HCs was significantly higher than that in epilepsy group (*P* < 0.01; Figures 2A,B). Venn diagram (Figure 2C) showed that the number of OTU shared by the two groups was 1184. In total, 2181 OTUs were unique to the epilepsy group, while only 65 OTUs were unique to the HC group, indicating that there were significant differences in microbial composition between the two groups.

### Differences in the Fecal Microbiome Between Patients With Epilepsy and Healthy Individuals

**FIGURE 3:**
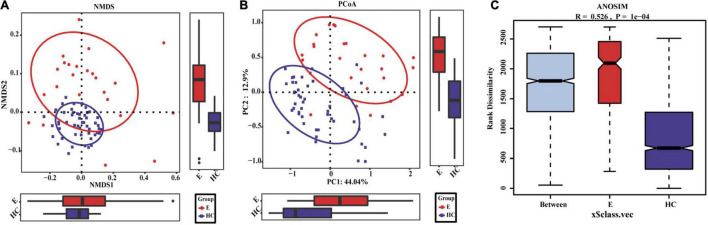
Differences in fecal microbiomes between Eps (*n* = 24) and HCs (*n* = 50). **(A)** β-Diversity was calculated by NMDS analysis of unweighted UniFrac distances. Samples from the Eps and HC (blue) groups were distinctly separated along the NMDS2 axis, which means that individuals with epilepsy were substantially different from healthy individuals. **(B)** PCoA of unweighted UniFrac PC1-2 showed that the samples from the Eps and HC (blue) groups were distinctly separated along the PC2 axis, which means that the overall fecal microbiota compositions were markedly different between Eps and HCs. **(C)** ANOSIM showed that there were significant differences between the two groups (*R* = 0.526, *P* = 1e-04). Each symbol represents a sample (red, Eps; blue, HCs). The variance explained by the PCs is indicated by parentheses on the axes.

**FIGURE 4:**
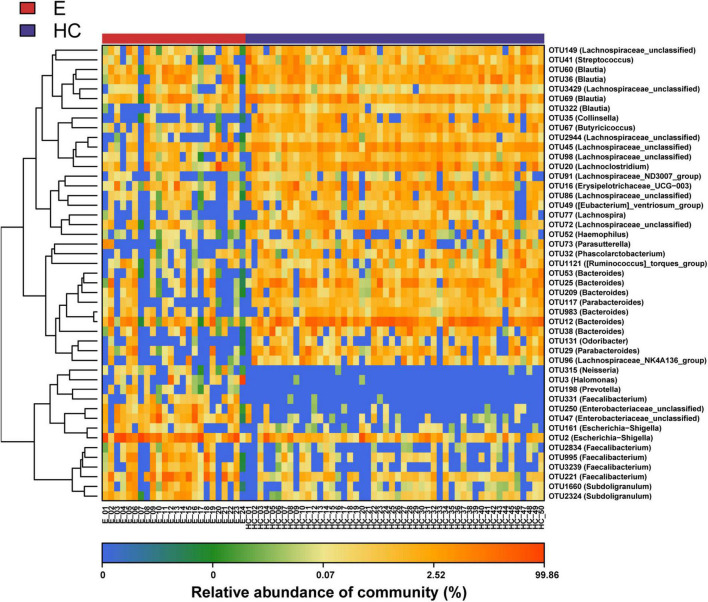
Heat map of the relative abundances of differential OTUs between Eps (*n* = 24) and HCs (*n* = 50). For each sample, the columns show relative abundance data for differential OTUs on the right. The relative abundance of each OTU was used to plot the heat map (blue, low abundance; red, high abundance). Group data are shown above the plot: HCs, right, blue line; Eps, left, red line. Each row represents one OTU. Eps, patients with epilepsy; HCs, healthy controls; NMDS, nonmetric multidimensional scaling; PCoA, principal coordinate analysis; ANOSIM, analysis of similarities; OTUs, operational taxonomic units.

Nonmetric multidimensional scaling analysis and PCoA based on the distribution of the OTUs were conducted to illustrate the microbiome space of different samples. β-diversity was mainly demonstrated by unweighted unifrac-based NMDS and PCoA. The fecal microbial communities in patients with EPs and HCs (blue) were separated along the NMDS2 axis (Figure 3A), indicating that the fecal microflora differed significantly between the two groups. In the PCoA diagram (Figure 3B), samples from the Eps and HC (blue) groups were separated on the PC2 axis, and the contribution rate of PC2 to sample separation was 12.9%, which also confirmed a significant difference in the fecal microbial community between the two groups. In the ANOSIM (Figure 3C), *R* = 0.526 and *P* = 1e-04, so the difference between the two groups was statistically significant. The microbial community heat map (Figure 4) showed that 14 OTUs, including *Subdoligranulum*, *Faecalibacterium*, *Enterobacteriaceae-unclassified* and *Escherichia-Shigella*, were more abundant in Eps than HCs. In contrast, 33 OTUs, including *Blautia*, *Lachnospiraceae-unclassified*, *Parabacteroides*, *Collinsella* and *Bacteroides*, were enriched in the HCs compared with Eps.

### Composition and Comparison of Fecal Microbiomes in Patients With Epilepsy and Healthy Controls

**FIGURE 5:**
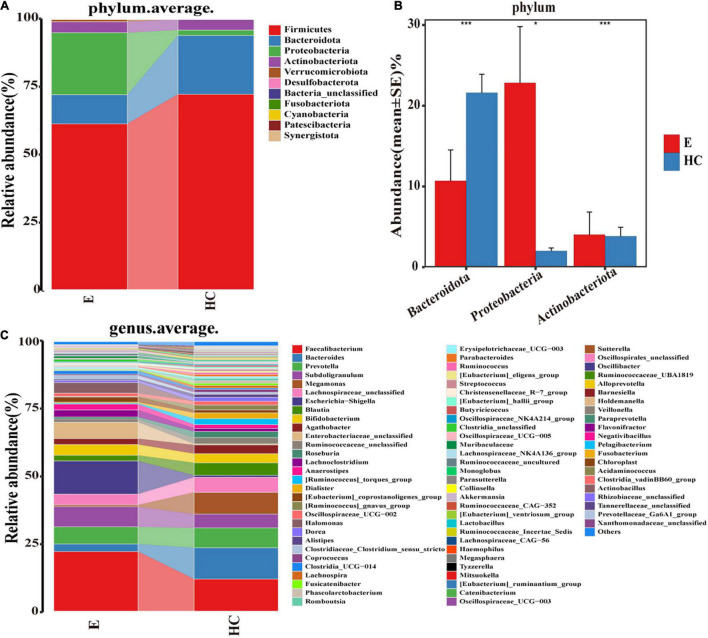
Composition and comparison of fecal microbiomes in Eps (*n* = 24) and HCs (*n* = 50). Composition of the fecal microbiota at the **(A)** phylum and **(C)** genus levels in Eps versus HCs. Comparison of the fecal microbiota at the **(B)** phylum between the two groups. Significant differences in the abundance of predominant genera between Eps and HCs (blue). The average abundance of each bacterium is depicted as the mean ± SE. *P*-values were calculated by a Wilcoxon rank-sum test and are shown in the Supplementary Data. *P < 0.05; ***P < 0.001; Eps, patients with epilepsy; HCs, healthy controls.

According to the OTU annotations of each fecal sample, the relative microbial abundance of all samples at the phylum, class, order, family and genus levels was calculated. A Wilcoxon rank-sum test was used to analyze the significant difference in microbial composition between groups. FDRs and *q* values were calculated for *P* (Cohen et al., 2019). The average compositions and relative abundances of the microbial community in the two groups at the phylum and genus levels are shown in Supplementary Figure 2. At the phylum level (Figure 5A), the bar plots revealed that the cumulative average proportion of *Firmicutes*, *Bacteroidota*, *Proteobacteria* and *Actinobacteriota* accounted for more than 98% in the two groups. There were no significant changes between the groups in terms of *Firmicutes* (*P* > 0.05). However, the relative abundance of *Bacteroidota* in the HCs was higher than that in the Eps, while those of the *Proteobacteria* and *Actinobacteriota* in the Eps was higher than those in the HCs (all *P* < 0.05, Figure 5B). The composition (Figure 5C) and comparison (Supplementary Figure 3D) of fecal microflora of the two groups at the genus level were also shown. At the genus level, *Bacteroides*, *Faecalibacterium*, *Megamonas*, *Prevotella, Lachnospiraceae-unclassified*, *Escherichia-Shigella*, and *Subdoligranulum* accounted for >50% on average in both groups (Supplementary Data 2–4).

At the class level, four bacterial groups, including *Bacteroidia, Negativicutes, Bacilli*, and *Coriobacteriia*, had a higher relative abundance in the HCs, while three bacterial groups, including *Gammaproteobacteria, Alphaproteobacteria*, and *Actinobacteria*, were enriched in the Eps. At the order level, 11 bacterial groups, including *Lachnospirales* and *Bacteroidales*, had a higher abundance in the HCs, whereas six bacterial groups, including *Enterobacteriales* and *Bifidobacteriales*, were enriched in the Eps. The fecal microorganisms in the two groups were also different at the family level, indicating that there were significant differences between the Eps and the HCs (Supplementary Data 5–8 and Supplementary Figures 3A–C).

### Phylogenetic Characteristics of the Fecal Microbial Communities in Epilepsy Patients

**FIGURE 6:**
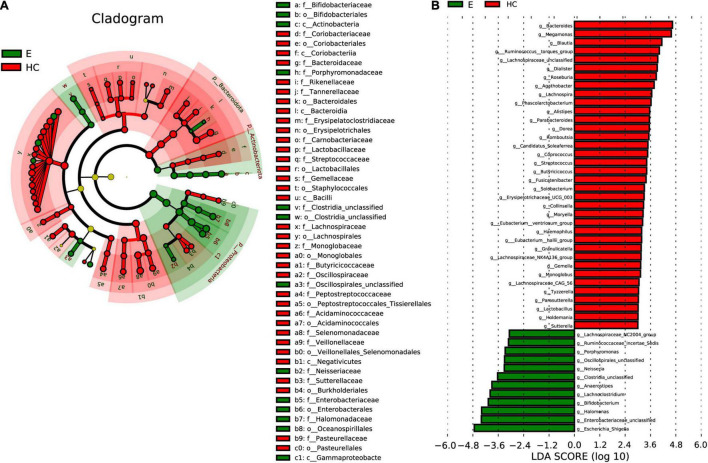
LEfSe analysis and LDA based on OTU characterizations of the microbiota in Eps (*n* = 24) and HCs (*n* = 50). **(A)** Cladogram generated by the LEfSe method showing the phylogenetic distribution of fecal microbiomes associated with Eps and HCs. Each filled circle represents one phylotype. Phylum and class are indicated by names on the cladogram. Order, family, and genus are listed in the right panel. Circle size is proportional to phylotype abundance. By default, the taxonomic levels are arranged outward from phylum to genus. Red circles on the branches represent microbial communities playing pivotal roles in epilepsy. Green circles represent microbial groups playing important roles in HCs. Yellow circles represent microbial groups of little significance in either group. The default criteria LDA > 3 and *P* < 0.05 indicate different species and a higher abundance in one group than in the other. **(B)** Histogram of LDA scores calculated for selected taxa showing significant differences in microbe type and abundance between Eps (green) and HCs. LDA scores on a log10 scale are indicated at the bottom. The significance of the microbial marker increases with the LDA score. Eps, patients with epilepsy; HCs, healthy controls; OTUs, operational taxonomic units; LEfSe, linear discriminant analysis effect size; LDA, linear discriminant analysis.

Linear discriminant analysis effect size analysis (Figure 6A) and the LDA genus score (Figure 6B) confirmed that 48 microbial biomarkers clearly distinguished Eps and HCs. Moreover, the divergence between the two groups was highly significant (*P* < 0.05). Biomarker names, LDA scores, log values and *P*-values are shown in Supplementary Data 9.

In the cladogram (Figure 6A) drawn by the LEfSe method, the phylogenetic distribution of the intestinal microbiota in patients with EPs (green) and HCs (Jandhyala et al., 2015) is shown. In the epilepsy group, microbes such as *c-Actinobacteria*, *c-Gammaproteobacteria*, *o-Bifidobacteriales*, *o-Clostridia-unclassified* and *f-Bifidobacteriaceae* had obvious advantages, while in the healthy group, microbes such as *c-Coriobacteriia*, *o-Bacteroidales* and *f-Rikenellaceae* had obvious advantages. The LDA score in Figure 6B shows that there was a significant difference in genera between Eps and HCs. Twelve genera, including *Escherichia-Shigella*, *Enterobacteriaceae-unclassified*, *Halomonas*, and *Bifidobacterium*, predominated in Eps, whereas 36 genera, including *Lachnospiraceae-unclassified*, *Bacteroides, Blautia* and *Lachnospira*, predominated in HCs (*P* < 0.05; LDA > 3).

### Gene Function Analysis

**FIGURE 7:**
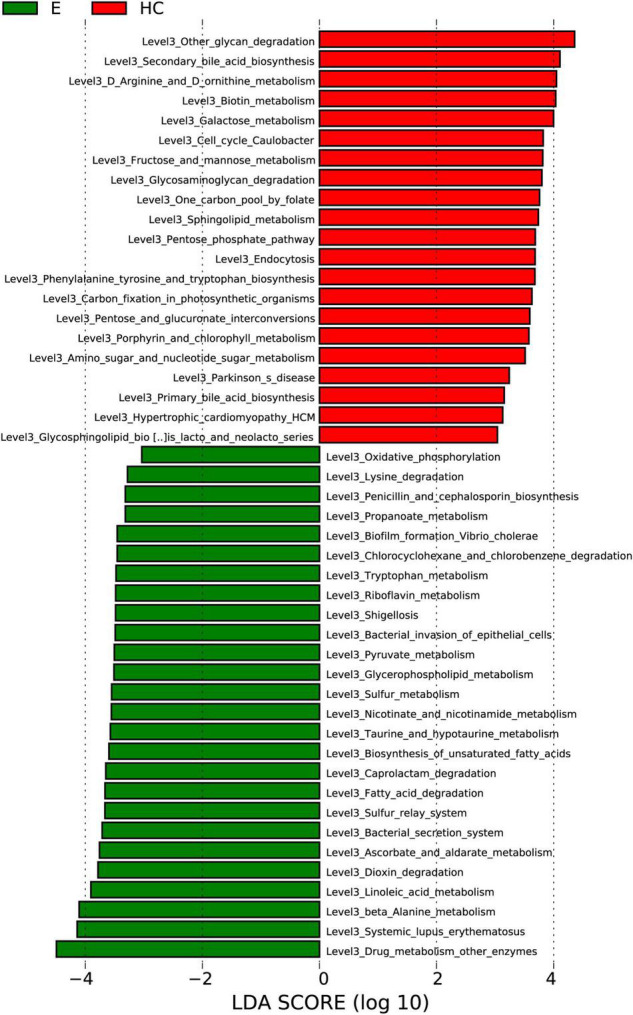
Functional analysis of predicted metagenomes. Differentially abundant KEGG pathways across Eps (*n* = 24) and HCs (*n* = 50) identified by the LEfSe method. Histogram of LDA scores calculated for selected pathways showing significant differences in gene functions between Eps (green) and HCs. LDA scores on a log10 scale are indicated at the bottom. The significance of the microbial marker increases with the LDA score. Eps, patients with epilepsy; HCs, healthy controls; OTUs, operational taxonomic units; LEfSe, linear discriminant analysis effect size; LDA, linear discriminant analysis.

To elucidate the functional and metabolic alterations of the intestinal microbiomes between the Eps and HC groups, metagenomes were next inferred from the 16S rRNA data, and the functional potential of the gut microbiota was analyzed. LEfSe analysis was used to identify the differential abundant KEGG pathways between the Eps (*n* = 24) and HCs (*n* = 50) (Figure 7). At level 3, 21 pathways, including other-glycan-degradation, secondary-bile-acid-biosynthesis and D-Arginine-and-D-ornithine-metabolism were enriched in the HCs (*P* < 0.05; LDA > 3). Twenty-six pathways, including oxidative-phosphorylation, drug-metabolism-other-enzymes, systemic-lupus-erythematosus, and beta-alanine-metabolism, were enriched in Eps (*P* < 0.05; LDA > 3).

### Correlations Between the Fecal Microbiome and Clinical Characteristics

**FIGURE 8:**
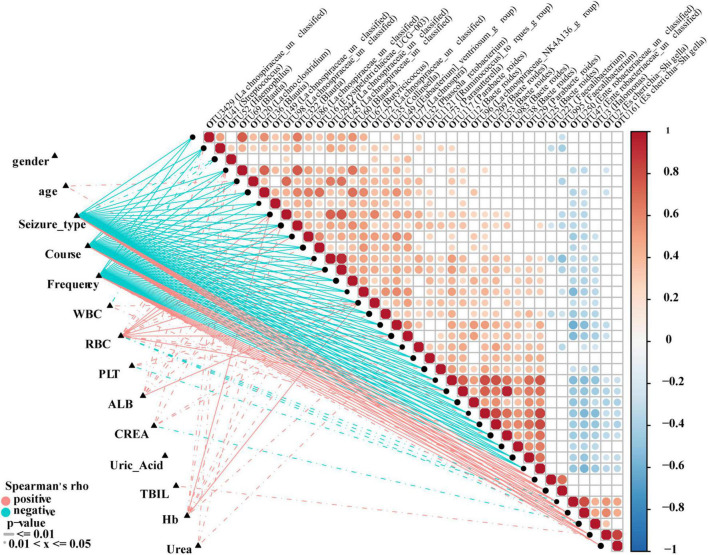
Correlation analysis of differential OTUs and clinical characteristics of epilepsy patients. Heat map showing partial Spearman’s correlation coefficients among 38 OTUs and clinical indices. Positive values indicate positive correlations. Negative values (blue) indicate inverse correlations. Solid lines represent *P* ≤ 0.01. Dotted lines represent 0.01 < *P* ≤ 0.05. The intensity of shading in circles is proportional to the magnitude of the association. Correlation direction was determined by Spearman’s method. Eps, patients with epilepsy; WBC, white blood cells; RBC, red blood cells; Hb, hemoglobin; PLT, platelets; ALB, albumin; UREA, urea; CERA, creatinine; UA, uric acid; TBIL, total bilirubin.

To analyze correlations between OTUs and the clinical characteristics of individuals with epilepsy, Spearman’s rank test was performed to consider potential drivers such as age, gender and course. The distance correlation plots in Figure 8 revealed partial Spearman correlation coefficients among 38 OTUs and 12 clinical indices, such as gender, seizure, course, frequency, and WBC. We used at least one correlation coefficient greater than 0.4 and *P* < 0.05 as the criteria for correlation. Seven of the clinical indicators had higher correlations with OTUs: Seizure-type, course and frequency are strongly correlated with all 38 OTUs, and negatively correlated with the same 31 OTUs, but positively correlated with the remaining seven OTUs. In addition, age, ALB, TBIL, Hb, and Urea were only positively correlated with part of OTUs. WBC, RBC, PLT, and CREA were positively correlated with some OTUs and negatively correlated with some OTUs. Interestingly, their related OTUs overlapped. And their negative correlation OTUs is the positive correlation OTUs of seizure-type, course and frequency. OTU69 (*Blautia*), OTU72 (*Lachnospiraceae-unclassified*), OTU98 (*Lachnospiraceae-unclassified*), and OTU322 (*Blautia*) are strongly correlated with more than half of the clinical indicators and may be the focus of research (Supplementary Data 10).

### Potential Use of Fecal Microbiome-Based Signatures in Epilepsy Diagnosis

**FIGURE 9:**
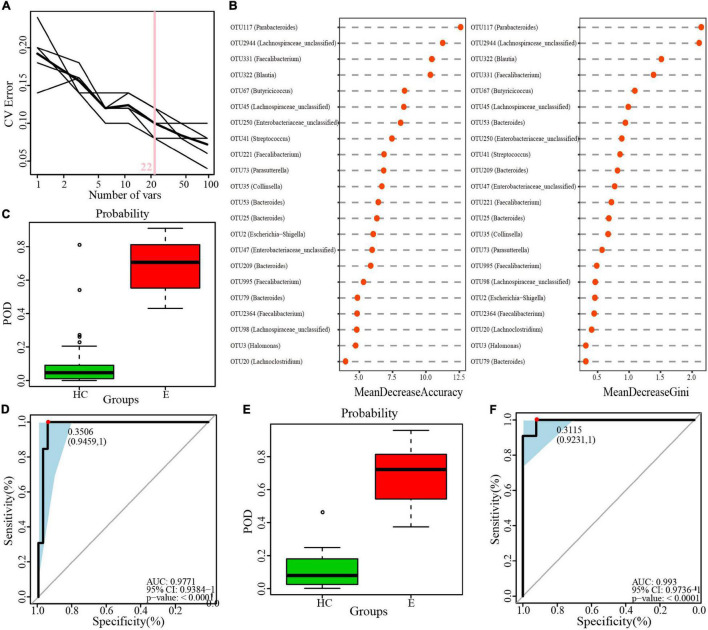
Identification of microbial OTU-based Ep markers by random forest models. **(A)** 22 OTUs were selected by random forest models as the optimal epilepsy biomarkers. **(B)** Importance distribution map of the selected microbial markers in the model. **(C)** POD was significantly higher in Eps than HCs in the training set. **(D)** The POD index had an AUC = 0.9771 with a 95% CI = 0.9384–1 between Eps and HCs in the training set. **(E)** POD was significantly higher in Eps than HCs in the test set. **(F)** The POD index had an AUC = 0.993 with a 95% CI = 0.9736–1 between Eps and HCs in the test set. Eps, patients with epilepsy; HCs, healthy controls; OTUs, operational taxonomic units; CV error, cross-validation error; POD, probability of disease; AUC, area under the curve.

A minimum OTU combination was identified to assess the potential of gut microbial markers as a noninvasive diagnostic tool for epilepsy. A cross-validation curve of the random forest model revealed 22 OTU biomarkers, namely, OTU2944 (*Lachnospiraceae-unclassified*), OTU117 (*Parabacteroides*), OTU3 (*Halomonas*), OTU45 (*Lachnospiraceae-unclassified*), OTU322 (*Blautia*) and OTU35 (*Collinsella*), etc. (Figure 9A and Supplementary Data 11). The mean decrease in accuracy and mean decrease in the Gini coefficient in the stochastic decision forest model showed the distribution of OTU importance (Figure 9B). The POD index was calculated using the microflora data and 22 OTU biomarkers and found to be markedly higher in the Eps group than in the HC group (Figures 9C,E). The ROC constructed with the training set showed that the AUC was 0.9771 and the 95% confidence interval was 0.9384–1 (*P* < 0.0001) (Figure 9D). For the test set, the AUC was 0.993, and the 95% confidence interval was 0.9736–1 (*P* < 0.001) (Figure 9F). These data validated the significant diagnostic potential of gut microbial markers for epilepsy.

## Discussion

Epilepsy is a complex condition with multiple risk factors and strong genetic predisposition, and more than 75% of people with active epilepsy go untreated in low- and middle-income countries, constituting a major treatment gap (Saxena and Li, 2017). Epilepsy is a major burden in terms of quality of life, morbidity and mortality risk. However, most of the studies on the relationship between intestinal microbiome and neurological diseases are focused on Alzheimer’s disease, Parkinson’s disease and autism, and there are few studies on the intestinal microecology of epilepsy. Reports of ketogenic diet therapy for epilepsy (Neal et al., 2008; Ang et al., 2020) have increased the focus of future research on the potential role of microbiota as a mediator of epilepsy. In our study, after rigorous screening, a total of 24 patients with EPs and 50 HCs were selected. We collected stool samples from the participants, sequenced the 16S rRNA and divided them into a training set and a validation set. The best biomarkers were sought and diagnostic model was constructed in the former, then validation was made in the test set. Our study is the first to elucidate the differences in gut microbiota between epileptic patients and healthy controls in central China. In the world to provide objective basis and support for the study of epilepsy and intestinal microbe. Although the number of participants was small, it injected its own strength into the ocean and provided evidence and guidance for more studies in the future.

To date, there have been only a few reports on epilepsy and intestinal microecology. Among these studies, Xie et al. (2017), which mainly considered intractable epilepsy in babies, revealed that the relative abundances of *Firmicutes* and *Proteobacteria* increased, while those of *Bacteroidetes* and *Actinobacteria* decreased. Most of the results of our study confirm those in previous reports, but there are some new findings that show some differences and advantages compared with the results of other similar studies. At the phylum level, compared to those in HCs, *Proteobacteria* and *Actinobacteriota* in the Eps were significantly increased, while *Bacteroidota* abundance was significantly increased in HCs. Twenty-three genera were enriched in the Eps while fifty-nine genera were increased in the HCs. We identified 22 microbial markers for the diagnosis of epilepsy. Moreover, the POD values indicated that the incidence in the epilepsy group significantly increased. The AUC for the training set reached 0.9771, and the AUC for the test set reached 0.993.

The differences in our study compared with others can be attributed to regional differences. Regional differences are the main factors affecting intestinal microecology, and the results may vary with different patients from different areas. In addition, the small sample size may also have a certain impact, and more trials are necessary. Exogenous factors can affect intestinal microbiome to increase seizures, such as diet, infection (Ahlers et al., 2019) and antibiotic use (Nørgaard et al., 2012), among which carbapenems (Cannon et al., 2014), imipenem, and meropenem (Owens, 2008; Leibovitch and Jacobson, 2015) are all highly associated with seizures. Although we excluded people with infections and those using probiotics and antibiotics when selecting subjects, there was no guarantee of diet or environmental effects.

By participating in the physiology and pathology of cellular organisms, microbiome exerts certain influence on disease and health. In terms of the nervous system, several studies have demonstrated the importance of the gut microbiome. In particular, the microbiota–gut–brain axis is proposed to link the microbiome with the intestine and brain as a whole, and explore the possible mechanisms and pathways. This two-way communication signal transmission mechanism is complex, including neural, endocrine, immune and metabolic pathways (Grenham et al., 2011; El Aidy et al., 2015). Ma et al. (2019) found that changes in the composition of intestinal flora contributed to inflammation by regulating innate immunity, especially NF-κB signaling, while restoring unbalanced intestinal flora alleviates symptoms (Sanz and Moya-Pérez, 2014; Jang H.M. et al., 2018; Jang S.E. et al., 2018). People who cut the vagus nerve, which connects the gut to the spinal cord, have a reduced risk of certain neurological conditions (Svensson et al., 2015). In addition, increased intestinal and blood-brain barrier permeability caused by dysbiosis of the microbiome may mediate or influence the onset of AD and other neurodegenerative diseases (Angelucci et al., 2019). Bacteria in the gut flora can secrete large amounts of amyloid and lipopolysaccharide, which may help regulate parts of signaling pathways and production of pro-inflammatory cytokines.

Our experiment revealed a difference in intestinal microecology between Eps and HCs in central China, and it was found that *Proteobacteria* and *Actinobacteriota* were significantly increased in the Eps, while *Bacteroidota* abundance was significantly increased in HCs. These results are convenient for diagnosis of epilepsy and targeted treatment of patients with epilepsy. However, our experiment also had some shortcomings. The sample size was insufficient. The only thing we can do is expand the sample size and try to improve the standardization of the sampling procedure to minimize the influence of other interference factors. Therefore, a more systematic study should be conducted on a large random sample of individuals from different regions and with different eating habits, genders and ages. Moreover, individual microbiology is associated with many confounding factors, such as time, diet, and the environment. This has been shown in multiple studies (Ren et al., 2019, 2020; Lou et al., 2020; Rao et al., 2020). In particular, Ang et al. (2020) conducted gene sequencing and metabolomics analysis on fecal samples and found that the structure and function of the intestinal microbial community changed significantly during ketogenesis, indicating that diet does interfere with intestinal microecology.

## Conclusion

In summary, our study highlighted the differences in the fecal microbiota between patients with EPs and HCs and analyzed them at the phylum and genus levels, revealing twenty-two biomarkers that distinguish patients with epilepsy from healthy subjects and thus providing a noninvasive method for diagnosing epilepsy.

## Data Availability Statement

The raw Illumina read data for all samples were deposited in the European Bioinformatics Institute European Nucleotide Archive database (accession number: PRJNA701117).

## Ethics Statement

The studies involving human participants were reviewed and approved by the Ethics Committee of Scientific Research and Clinical Trial, The First Affiliated Hospital of Zhengzhou University (No. 2021-KY-0574-002). Written informed consent to participate in this study was provided by the participants’ legal guardian/next of kin.

## Author Contributions

YJ and YL designed the study. GC, ZL, YC, TW, JL, HW, YZ, and YS collected clinical samples. SL and BR extracted the bacterial DNA. ZR and SL completed data analysis. SL and GC wrote the manuscript. All authors reviewed and approved the manuscript.

## Conflict of Interest

The authors declare that the research was conducted in the absence of any commercial or financial relationships that could be construed as a potential conflict of interest.

## Publisher’s Note

All claims expressed in this article are solely those of the authors and do not necessarily represent those of their affiliated organizations, or those of the publisher, the editors and the reviewers. Any product that may be evaluated in this article, or claim that may be made by its manufacturer, is not guaranteed or endorsed by the publisher.

## Footnotes

1
http://drive5.com/uchime/gold.fa

2
http://www.R-project.org/

3
www.microbesonline.org/programmers.html

## Abbreviations

OTU: operational taxonomic unit
Eps: epilepsy patients
HCs: healthy controls
ILAE: International Anti-Epilepsy Alliance
PCR: polymerase chain reaction
NMDS: nonmetric multidimensional scaling
PCoA: principal coordinate analysis
LDA: linear discriminant analysis
LEfSe: linear discriminant analysis effect size
KEGG: Kyoto Encyclopedia of Genes and Genomes
COG: clusters of orthologous groups
HUMAnN: HMP unified metabolic analysis network
SD: standard deviation
ROC: receiver operating characteristic curve
POD: probability of disease
AUC: area under the curve
ANOSIM: analysis of similarities
FDRs: false discovery rates
WBC: white blood cells
RBC: red blood cells
PLT: platelets
ALB: albumin
CREA: creatinine
UA: uric acid
Hb: hemoglobin
UREA: urea
EEG: electroencephalogram
TBIL: total bilirubin.
