# Supplementary material for: Gut Microbiome Distinguishes Patients With Epilepsy From Healthy Individuals
Source: Front Microbiol. 2022 Jan 7;12:696632. doi: 10.3389/fmicb.2021.696632 (PMC8777111; doi:10.3389/fmicb.2021.696632)
Supplement: Supplementary file 1 [file Data_Sheet_1.docx]

**Supplementary Figures**

**
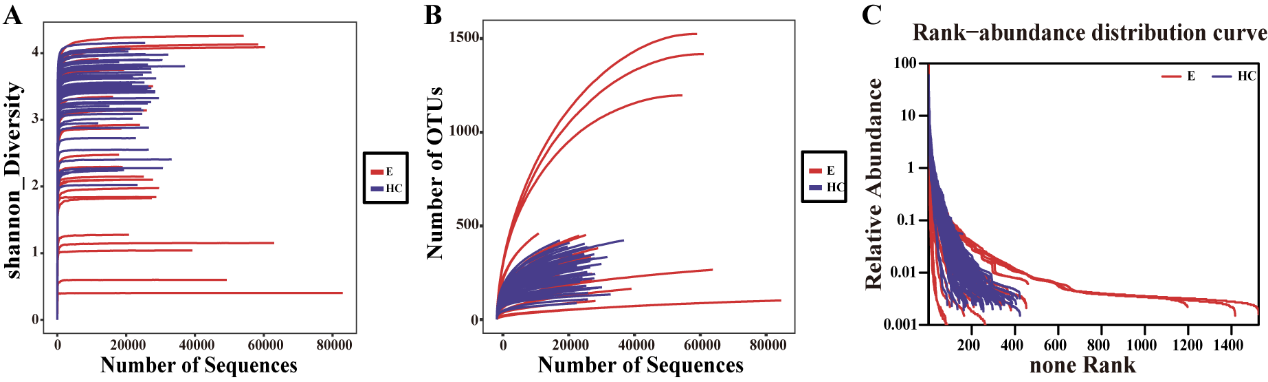
**

**Figure S1** Data quality of bacterial 16S rRNA gene sequences. (**A**) Shannon-Wiener curve showed that estimated OTUs richness basically approached saturation in Eps (n = 24) (red) and HCs (n = 50) (blue). (**B**) Rarefaction analysis and (**C**) Rank-Abundance curve, showing that this study needs more samples of epilepsy patients. Estimated OTU richness approached saturation in samples of healthy controls, while the curve in epilepsy group was still steep. Ep, epilepsy; HCs, healthy controls; OTUs, Operational Taxonomy Units.


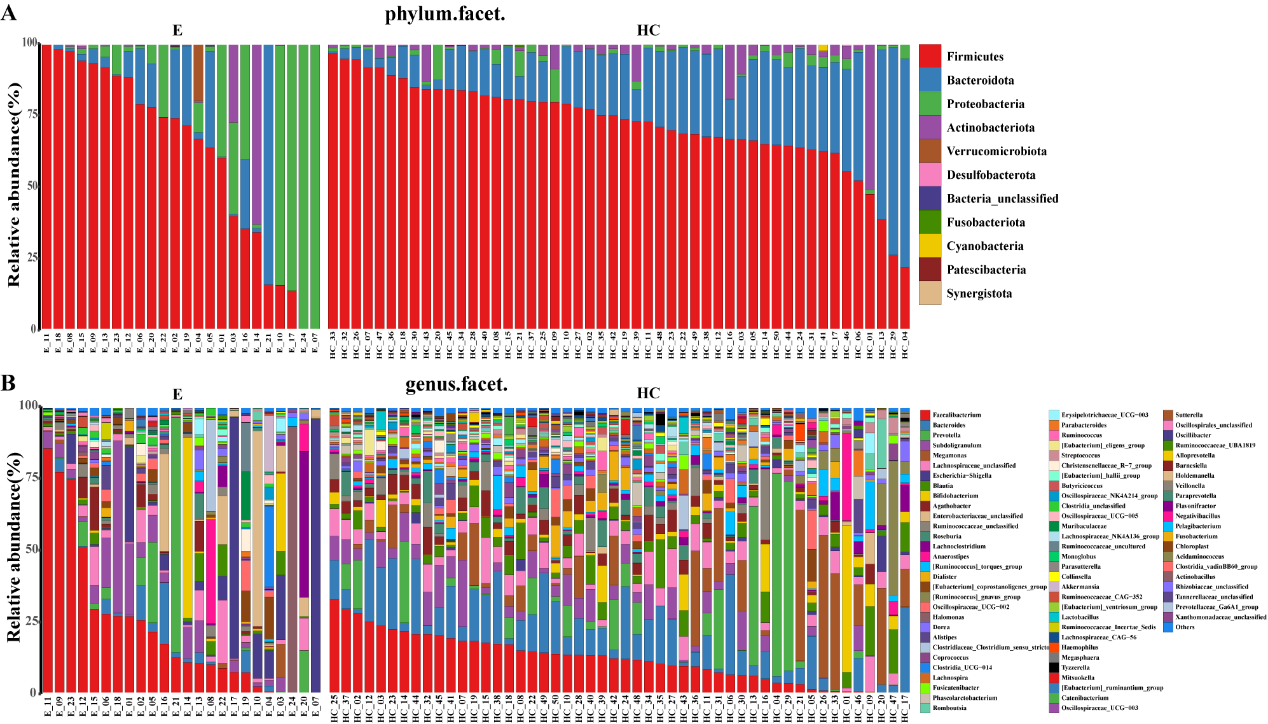


**Figure S2** Composition of fecal microbiota on taxonomic level between Eps (n = 24) and HCs (n = 50). The microbial community bar plot of Eps and HCs visually reflected the relative abundance of microbiome at (**A**) phylum level and (**B**) genus level for each sample. Eps, epilepsy; HCs, healthy controls.

**
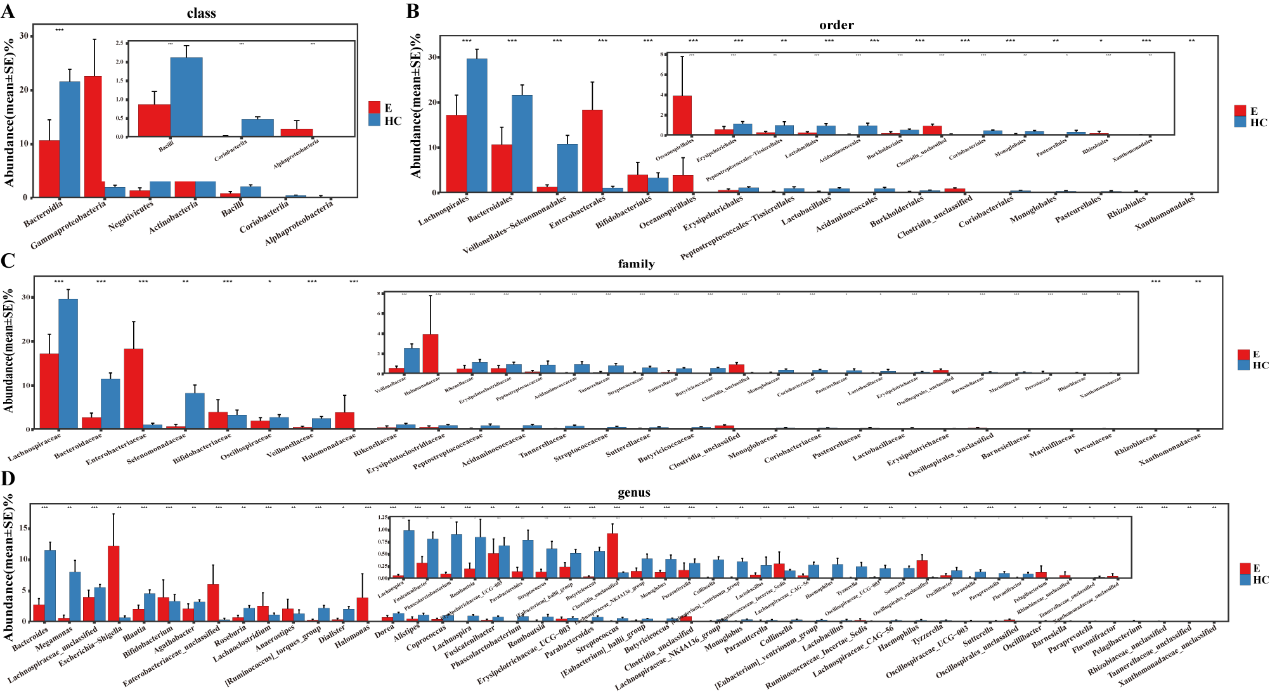
**

**Figure S3** Comparison of fecal microbiome on taxonomic level between Eps (n = 24) and HCs (n = 50). Comparison of fecal microbiota at (**A**) the class level, (**B**)

order level, (**C**) family level and (**D**) genus level between Eps (red) and HCs (blue). The average abundance values for each bacterium were depicted as mean ± SE. *P* values were calculated using the Wilcoxon rank sum test, and are shown in Supplementary Data. Significant differences by **P* < 0.05; ***P* < 0.01, and ****P* < 0.001. Eps, epilepsy; HCs, healthy controls.
